# Flavones Contents in Extracts from *Oroxylum indicum* Seeds and Plant Tissue Cultures

**DOI:** 10.3390/molecules25071545

**Published:** 2020-03-28

**Authors:** Piyanuch Rojsanga, Somnuk Bunsupa, Pongtip Sithisarn

**Affiliations:** 1 Department of Pharmaceutical Chemistry, Faculty of Pharmacy, Mahidol University, Bangkok 10400, Thailand; piyanuch.roj@mahidol.ac.th; 2 Department of Pharmacognosy, Faculty. of Pharmacy, Mahidol University, Bangkok 10400, Thailand; somnuk.bun@mahidol.ac.th

**Keywords:** oroxylum indicum, baicalin, baicalein, chrysin, HPLC

## Abstract

*Oroxylum indicum* (L.) Benth. ex Kurz or Pheka, is a plant in the Bignoniaceae family with various traditional uses. The mature fruits promote anti-helminthic and stomachic effects, while the seeds have been used as a purgative and for the relief of tonsil pain. The young fruits are popularly consumed as vegetables, while the seeds are one of the components in traditional drink formulations. To develop new plant raw material sources, a plant tissue culture technique was used to generate plant tissue cultured samples from the seeds of *O. indicum*. Plant tissue cultured samples were collected from three different growth stages; 4 days, then at 3 and 9 weeks, and prepared as crude extracts by maceration with ethanol, along with the seed raw material sample. A high performance liquid chromatographic (HPLC) method was used for quantitative analysis of the contents of the three major flavones; baicalin, baicalein, and chrysin in the extracts from the seeds and plant tissue cultured samples of this plant. Baicalin was found in the highest amount among these three flavones in all extracts. The seed extract contained the highest baicalin content (24.24% *w*/*w* in the extract), followed by the shoot extract from tissue-cultured plant at week 3 (14.78% *w*/*w* of the extract). The amounts of chrysin in all *O. indicum* showed the same trend as the contents of baicalin, but the amounts were lower, while baicalein was accumulated at the lowest amount among three flavonoids and the amounts were quite stable in all *O. indicum* extracts. From the results, *O. indicum* seed and plant tissue cultured extracts have potential as sources of flavones, which could be further developed as health products in the future.

## 1. Introduction

*Oroxylum indicum* (L.) Vent. or pheka is a plant in the Bignoniaceae family. Its various parts have been traditionally used for many medicinal purposes, including anti-helminthic and stomachic effects, and for the relief of tonsil pain [1,2]. The pods, seeds, and root bark of this plant contain many flavonoids such as baicalein, biochanin A, oroxylin A, chrysin, apigenin, and their glycosides [3,4,5,6,7,8,9]. Some alkaloids and triterpenes were found in the seeds and pods [5,10], while the seed oil contained some fatty acids such as lauric, myristic, palmitic, stearic, oleic, and linoleic acids [1]. Some phenolics such as anthraquinone, tannic acid and ellagic acid, alkaloids, and some phytosterols were also reported in the leaves [3,4,11,12,13]. From our previous reports, ethanol extracts from *O. indicum* fruits and seeds exhibited in vitro antibacterial activities against clinically isolated *Staphylococcus intermedius* and *Streptococcus suis*, and showed *in vitro* antioxidant activity tested by the DPPH scavenging method [14]. The main flavones in the seeds and fruit extracts, analyzed by HPLC, were found to be baicalin, followed by baicalein and chrysin [15]. Extracts from other plant parts of *O. indicum*, including the leaves, flowers, seeds, stalks, and tissue-cultured plants and callus were also studied, and it was found that most extracts exhibited high *in vitro* antioxidant activities, determined by a DPPH scavenging assay, with high total phenolic and total flavonoid contents. Phytochemical analysis by TLC and LC-MS showed that each extract from the different plant parts had specific chromatographic fingerprints, with the presence of baicalein and chrysin as major compounds as well [15]. A plant tissue culture technique was used to increase and develop new sources of *O. Indicum* raw materials. It was found that extracts from tissue-cultured plants in the root growing stage, cotyledon growing stage, and leaf growing stages and extracts from callus cultures contained the highest amounts of total phenolic and total flavonoid content [16]. Therefore, this experiment was set up in order to quantitatively analyze the three major flavone contents in extracts from *O. indicum* tissue-cultured plant samples using the high-performance liquid chromatographic (HPLC) method, which was previously optimized and validated [15].

## 2. Results

**Figure 1:**
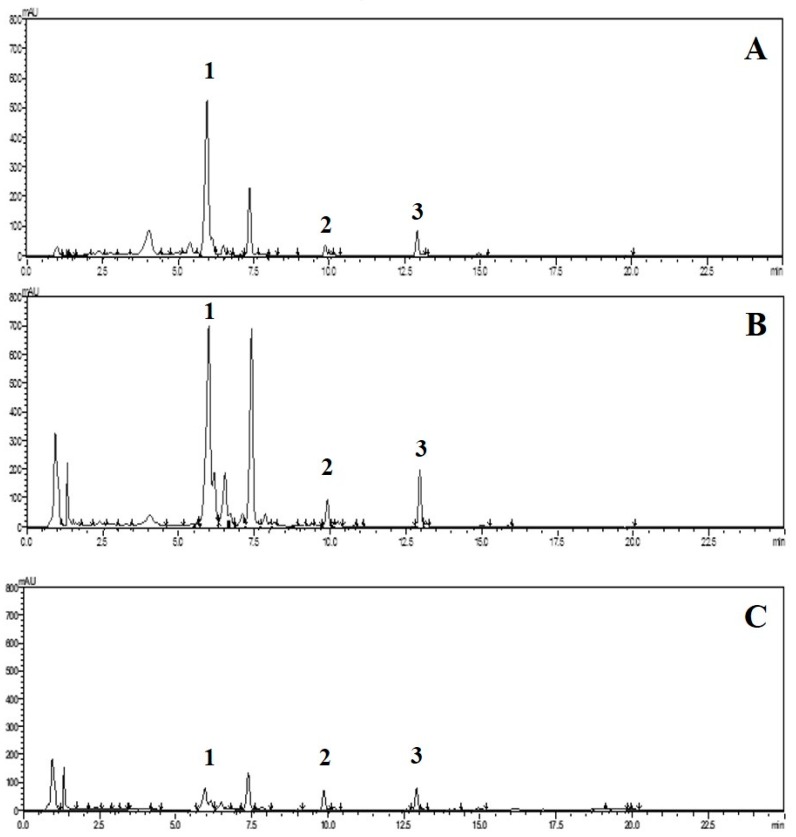
High-performance liquid chromatographic (HPLC) chromatograms of *Oroxylum indicum* extracts; **A** = seed extract (RAW), **B** = tissue-cultured plant shoot week 3 extract (TW3S), **C** = tissue-cultured plant root week 3 extract (TW3R). HPLC peaks; 1 = baicalin, 2 = baicalein, 3 = chrysin.

It was found that all extracts from *O. indicum* showed peaks that corresponded to baicalin, baicalein, and chrysin at retention times of 6.36, 10.10, and 13.01 min, respectively. Linear correlations from 1.02–102, 1–100, and 0.99–99.12 μg/mL were obtained from baicalin, baicalein, and chrysin, respectively. The *r* values for all three compounds were > 0.9990, confirming the linearity of the method. From the investigation of peak purity, there was no indication of co-elution or impurities from all peaks of interest. The recoveries of all three concentrations of all compounds were close to theoretical amounts (% recoveries were in the range from 94% to 96%, %RSD < 2). Percentages of relative standard deviation (%RSD) of six standard baicalin, baicalein, and chrysin solutions analyzed on three different days (intermediate precision) were less than 2%, while the %RSD values for repeatability were not more than 2%. Therefore, the method can be regarded as precise. The validation parameters obtained from the optimized HPLC methods were acceptable [16]; therefore, this analytical HPLC method could be used for quantitative analysis of the three flavonoids in the *O. indicum* extracts. For system suitability parameters, tailing factors of all three standard compounds were less than 2, theoretical plate numbers were in the range of 10,000 to 90,000, %RSD values of the peak area were less than 2, and the standard deviation (SD) values of the retention times of each peak were less than 1, suggesting the suitability of the analytical method. Figure 1 shows HPLC chromatograms of *O. indicum* seed and tissue-cultured plant shoot and root in week 3 extracts.

From Table 1, the extract from *O. indicum* seeds (RAW) contained the highest amount of baicalin (24.24 ± 2.44% *w*/*w* in the extract), followed by the extract from the *O. indicum* tissue-cultured plant shoot in week 3 (TW3S), and the extract from the *O. indicum* tissue-cultured plant on day 4 (TD4) (14.78 ± 0.28 and 10.67 ± 0.58% *w*/*w* in the extract, respectively). The amounts of baicalin in these three extracts are higher than 10% *w*/*w*. The amounts of chrysin in *O. indicum* extracts showed the same trend to those of baicalin, but TD4 contained the highest amount (3.60 ± 0.21% *w*/*w* in the extract), followed by RAW and TW3S (2.43 ± 0.04 and 1.96 ± 0.06% *w*/*w* in the extract, respectively). The shoot extract of tissue-cultured plant shoot week 9 (TW9S) contained the highest amount of baicalein (1.02 ± 0.01% *w*/*w* in the extract) and moderate amounts of baicalin and chrysin (3.18 ± 0.09, and 1.50 ± 0.05% *w*/*w* in the extract, respectively). The root extracts from tissue-cultured plant in weeks 3 and 9 contained a low flavonoids contents (lower than 1% *w*/*w* in the extract).

## 3. Discussion

Our previous study demonstrated that baicalin, baicalein, and chrysin were the major flavonoids in *O*. *indicum* seed and whole fruit extracts [15]. The seed ethanol and water extracts from *O*. *indicum* contained higher amounts of baicalin and chrysin contents than the young fruit ethanol and water extracts, while baicalein was found at around 1% *w*/*w* in the ethanol and water extracts of the seeds and in ethanol extract of young fruits, but was found in lower amounts in young fruit water extracts [15]. The young fruit ethanol extract from the Nakhon Pathom province was reported to promote strong antimicrobial activity against four clinical pathogenic bacteria, including *Staphylococcus intermedius*, *Streptococcus suis*, *Pseudomonas aeruginosa*, and *β-Escherichia coli* determined by the broth micro-dilution method [15]. Baicalin, baicalein, and chrysin exhibited antibacterial effects on the tested bacteria, in which baicalin promoted the highest inhibitory effects, suggesting it is the main active compound [15]. These three flavonoids were also tested for DPPH scavenging effects, in which baicalin and baicalein showed strong free radical scavenging activities, while chrysin promoted a low inhibitory effect [17]. The explanation about the lower antibacterial effects of the seeds ethanol and water extracts of *O*. *indicum*, which contained higher baicalin contents, but promoted lower antibacterial effects than the young fruit ethanol extract that contain lower baicalin content, was that the fixed oil in the seeds of *O*. *indicum* could interfere with the antibacterial effect of the seed extracts [15,18]. Extracts from *O. indicum* tissue-cultured plants in early stages (day 5, day 7, week 2, and week 3) were reported to contain high total phenolic contents (higher than 5 g% GAE) with moderate amounts of total flavonoids (around 1–2 g% QE), while extracts from the late stages of tissue-cultured plants (week 4–week 8) promoted lower total phenolic and total flavonoid contents (1–4 g% GAE, and cannot be detected −3 g% QE, respectively) [17]. In our current study, the root of the tissue-cultured plant was developed at day 4, while the cotyledons and true leaves were developed at weeks 3 and 9, respectively. The results corresponded to our previous study, which suggested that the tissue-cultured plant contained the highest total phenolic and total flavonoid contents during the development of the root in early stages (around day 5–week 3), and was still high in content when the cotyledons were developed (week 4–week 5). However, the contents of total phenolic and flavonoids decreases when the true leaves were developed, and the root parts of the tissue-cultured plants contained low amounts of total phenolic and total flavonoid contents [17]. Bergquist et al. suggested that flavonoid content in plants varies by the factors including genotype, environmental growing conditions, growth stage, postharvest handling, and storage conditions [19,20,21,22]. These factors could affect both the concentration of total flavonoids and the composition of flavonoid in the plants [19,22,23]. There were also studies that confirmed that flavonoid contents and compositions could be changed during plant growth [19,24,25,26,27]. There was a study that mentioned that the number of flavonoid compounds increased according to the subsequent growth phase, and there was no flavonoid detected in the root during the vegetative phase of the plant [23]. A study also suggested that pyracanthoside and rutin are two flavonoids that first appear in plantlets during the growth phases of *Pyracantha coccinea* [24]. Flavones, flavanones with or without only para oxygen substitute in the B ring, chalcones, and three-dihydrochalcones were reported to be present in the root of the plant during the reproductive phase, while 5,7-dihydroxy flavonols and flavanones with mono-, di-, and tri-oxy substitution of the B ring were found in the aerial parts [24]. Moreover, it was reported that the O- and C-glycosylation with one or two sugar units (glucose, galactose, and/or rhamnose) are usually found in the aerial parts, while in the root, only 5- or 7-O-monoglusoside were found [24]. Some studies also suggested that the accumulation of flavonoids in different plant parts, such as the leaves, flowers, and fruits is the result of the *in situ* biosynthesis and the translocation process from the leaves to other organs [25]. Flavonoid contents in different plant organs could be affected by this synthesis/transport relationship and the variation of the content could occur during plant organ development [28,29].

From our study, it was clearly shown that flavones are the major flavonoid in the seeds and tissue-cultured plants of *O. indicum*. Baicalin, baicalein, and chrysin were found to be the three main compounds. Baicalin was accumulated in the highest content in the mature seeds (24% *w*/*w* of the extract), and still remained in high contents in the aerial parts of the tissue-cultured plant until the cotyledons were produced (higher than 10% *w*/*w* of the extract), then the content decreased when the true leaves started to grow. Baicalein contents in the seeds and aerial parts of tissue-cultured plant from different growth stages are rather constant (around 1% *w*/*w* of the extract), while chrysin contents showed the same trend to baicalin contents, but the amounts of chrysin in the seeds and all aerial parts of tissue-cultured plant from different growth stages are lower than the baicalin contents in the extracts from the same plant parts (around 2–4% *w*/*w* of the extract). There were very small amounts of these three flavonoids accumulated in the root parts of tissue-cultured plants.

## 4. Materials and Methods

### 4.1. Chemicals

Deionized water was obtained by using a water purification system from Thermo Scientific Co. (Waltham, MA, USA). Standard baicalin and chrysin, at a pharmaceutical grade, were purchased from TRC (TRC, North York, ON, Canada)), and baicalein, at an analytical reference grade, was purchased from Sigma-Aldrich (St. Louis, MO, USA). Acetonitrile was purchased from Sigma-Aldrich (St. Louis, MO, USA) and phosphoric acid was purchased from Merck (Darmstadt, Germany).

### 4.2. Plant Materials

**Figure 2:**
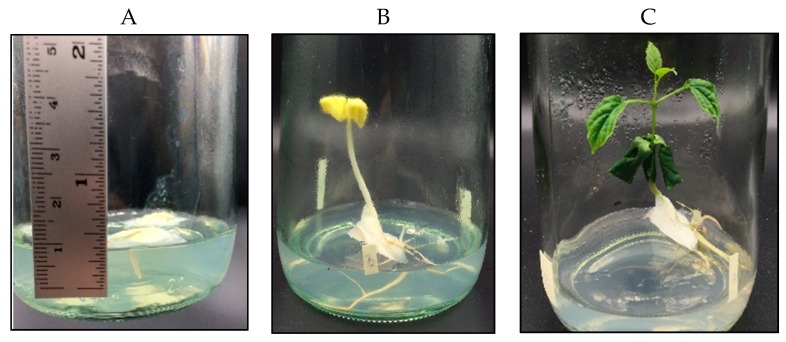
Physical characteristics of *Oroxylum indicum* tissue-cultured plant samples; **A** = day 4, **B** = week 3, **C** = week 9.

The mature seeds of *O. indicum* were collected from Lampang province in 2019. The plant samples were identified by Associate Prof. Pongtip Sithisarn. Some parts of the seeds were cleaned and dried in a hot air oven (Memmert, Schwabach, Germany) at 60 °C for 12 h, then ground using an electric milling machine (Ika-Werke, Staufen, Germany) (20 mesh sieve). Other parts of the seeds were prepared as described in a previous experiment to yield tissue-cultured plants at different growth stages [17]. Tissue-cultured plant samples were randomized using True Random Number Service software (Randomness and Integrity Services Ltd., Dublin, Ireland) at day 4, then week 3 and week 9. Tissue-cultured plant samples from week 3 and week 9 were separated for the aerial and root parts. Plant samples were cleaned and dried in a hot air oven at 60 °C for 12 h, then ground using an electric mill. Figure 2 shows the physical characteristics of the *O. indicum* tissue-cultured plant samples.

### 4.3. Plant Extract Preparations

Each sample of *O. indicum* seeds and tissue-cultured plant powders was separately extracted by maceration using 95% ethanol (plant:solvent ratio 1:20 *w*/*v*). Each powdered plant sample was separately macerated with 95% ethanol, using an electric flask shaker (Wisd Laboratory Instruments, Wertheim, Germany) for 6 h. The extraction solution was filtered after it was stored for 12 h. Each extraction process was repeated three times. The extraction solutions were then combined, filtered, and evaporated using a water bath to yield the dried extracts.

### 4.4. Analysis of Flavone Contents in O. indicum Extracts by HPLC

The contents of three flavones in the *O. indicum* extracts were quantitatively analyzed using the HPLC method modified from the previous report [15]. A high-performance liquid chromatographic system was performed using a Shimadzu LC-10ADVP system (Kyoto, Japan) equipped with a diode array detector (DAD) SPD-M10AVP and a column heater (Shimadzu, Kyoto, Japan). An X-terra C18 column (150 mm × 3.9 mm, 5 μm particle size) from Waters (Milford, MA, USA) was used. Gradient elution was performed with water –0.01% phosphoric acid (solvent A) and acetonitrile (solvent B) at a constant flow rate of 1.2 mL/min. Column temperature was 40 °C with an injection volume of 20 μL; detection was performed at 285 nm. Method validation was performed to confirm the system reliability. Linearity for standard baicalin, baicalein, and chrysin was determined by analysis of seven different concentrations, each injected in triplicate. Peak purity was investigated using a DAD for all peaks of interest. The precision of the method was evaluated by analyzing six independently prepared solutions of extracts from *O. indicum* fruit collected from the Chiang Rai province (3 mg/mL) on two different days. The peak area of each standard compound was determined, and the relative standard deviation percent (%RSD) was calculated. The accuracy of the method was confirmed by the determination of the recovery. The recovery of standard compounds was performed on extracts from *O. indicum* fruit collected from the Chiang Rai province (3 mg/mL), spiked with three concentrations of each compound (10, 20, and 30 μg/mL). The validated HPLC analytical method was applied to undertake a quantitative analysis of baicalin, baicalein, and chrysin in the extracts from the *O. indicum* samples. System suitability of the analytical method, including the tailing factor, theoretical plate, %RSD of the peak area, and the SD of the retention time, was also investigated.

## 5. Conclusions

Baicalin, baicalein, and chrysin are major flavones in extracts from the seeds and tissue-cultured plants from different growth stages of *O. indicum*. Baicalin, baicalein, and chrysin were quantitatively analyzed by a validated HPLC method. Among three flavonoids, baicalin was found in the highest concentrations in all extracts, but it was accumulated at the highest level in the extract from the seeds. The baicalin content remained high during the developments of the root and cotyledons of the tissue-cultured plants, but decreased when the growth of true leaves started. The amounts of chrysin in all *O. indicum* showed the same trend as to the contents of baicalin, but the amounts were lower than the amounts of baicalin. Baicalein was accumulated at the lowest amount among the three flavonoids, and the amounts were quite stable in all *O. indicum* extracts. From the results, tissue-cultured plants from *O. indicum*, especially in the stages before the growth of true leaves, could be sources of flavonoids for further pharmaceutical and medicinal applications and development.

## Figures and Tables

**Table 1:** Flavonoid contents in *O. indicum* extracts determined by high-performance liquid chromatographic (HPLC).

Sample	Content (% *w*/*w* in the Extract) *		
	Baicalin	Baicalein	Chrysin
RAW	24.24 ± 2.44 ^a^	0.72 ± 0.00 ^a^	2.43 ± 0.04 ^a^
TD4	10.67 ± 0.58 ^b^	1.02 ± 0.01 ^b^	3.60 ± 0.21 ^b^
TW3R	0.17 ± 0.00 ^c^	0.07 ± 0.00 ^c^	0.04 ± 0.00 ^c^
TW3S	14.78 ± 0.28 ^d^	0.79 ± 0.01 ^d^	1.96 ± 0.06 ^d^
TW9R	0.80 ± 0.02 ^e^	0.24 ± 0.02 ^e^	0.12 ± 0.00 ^e^
TW9S	3.18 ± 0.09 ^f^	1.09 ± 0.01 ^f^	1.50 ± 0.05 ^f^

* Different letters in the same column are significantly different (*p* < 0.05). RAW, extract from *O*. *indicum* seeds; TD4, extract from *O*. *indicum* tissue-cultured plant on day 4; TW3R, extract from *O*. *indicum* tissue-cultured plant root in week 3; TW3S, extract from *O*. *indicum* tissue-cultured plant shoot week 3; TW9R, extract from *O*. *indicum* tissue-cultured plant root in week 9; TW9S, extract from *O*. *indicum* tissue-cultured plant shoot in week 9.
